# Investigation of Quasi-Static Indentation Response of Inkjet Printed Sandwich Structures under Various Indenter Geometries

**DOI:** 10.3390/ma10030290

**Published:** 2017-03-14

**Authors:** Vishwesh Dikshit, Arun Prasanth Nagalingam, Yee Ling Yap, Swee Leong Sing, Wai Yee Yeong, Jun Wei

**Affiliations:** 1 Singapore Centre for 3D Printing, School of Mechanical and Aerospace Engineering, Nanyang Technological University, 50 Nanyang Avenue, Singapore 639798, Singapore; vishdixit@ntu.edu.sg (V.D.); NA0003TH@e.ntu.edu.sg (A.P.N.); YLYap@ntu.edu.sg (Y.L.Y.); SLSing@ntu.edu.sg (S.L.S.); 2 Singapore Institute of Manufacturing Technology, 71 Nanyang Drive, Singapore 638075, Singapore; jwei@SIMTech.a-star.edu.sg

**Keywords:** three-dimensional (3D) printing, additive manufacturing, quasi-static indentation, brittle fracture, cracks, failure mechanism, energy absorption, sandwich structure, damage

## Abstract

The objective of this investigation was to determine the quasi-static indentation response and failure mode in three-dimensional (3D) printed trapezoidal core structures, and to characterize the energy absorbed by the structures. In this work, the trapezoidal sandwich structure was designed in the following two ways. Firstly, the trapezoidal core along with its facesheet was 3D printed as a single element comprising a single material for both core and facesheet (type A); Secondly, the trapezoidal core along with facesheet was 3D printed, but with variation in facesheet materials (type B). Quasi-static indentation was carried out using three different indenters, namely standard hemispherical, conical, and flat indenters. Acoustic emission (AE) technique was used to capture brittle cracking in the specimens during indentation. The major failure modes were found to be brittle failure and quasi-brittle fractures. The measured indentation energy was at a maximum when using a conical indenter at 9.40 J and 9.66 J and was at a minimum when using a hemispherical indenter at 6.87 J and 8.82 J for type A and type B series specimens respectively. The observed maximum indenter displacements at failure were the effect of material variations and composite configurations in the facesheet.

## 1. Introduction

Sandwich panels are widely used in the aerospace industries due to their light weight and excellent strength-to-weight ratios. These sandwich panels are prone to impact and indentation from loading of field vehicles, grits, hail, bird strike and are mainly found in aircraft bodys, aircraft wings, and nacelles. These impacts and indentations lead to huge damage and drop in the performance of sandwich panels. Hence to overcome this, sandwich panels are designed by varying the core design, height, thickness, cell density etc. Conventional honeycomb and cellular core structures are manufactured from materials like paperboard, aluminum, polypropylene etc. These structures are engineered using gear presses, extrusion from blocks of extruded profiles and then via expansion and corrugation processes. Sandwich cores are also made up of composites like glass, Kevlar, carbon and Nomex aramid fiber. Efforts are also made to improve the performance by introducing multilayer [1] and fibre facesheets [2].

Core design plays a major role in determining the impact, indentation resistance and load bearing capacity of sandwich panels [3]. Thus, to improve the load bearing characteristics of sandwich panels, an efficient core design is necessary. Kirigami structures used as sandwich cores are modified to improve the energy absorption characteristics. An investigation modifying the Kirigami non-miura folded structures was done, based on the cube and egg box tessellated patterns. Results showed 41% improvement in energy absorption compared to conventional Kirigami cores [4]. Experimental studies on the flatwise compressive behavior of newly designed lattice cores were performed to improve the compressive strength of sandwich structures. In this investigation, the lattice patterns were waterjet cut from paper boards and vacuum assisted resin transfer molding (VARTM) technique was used to laminate the facesheet with the core. This technique of layering the facesheet improved the compressive strength [5]. Impact studies on trapezoidal structures with multilayer core and facesheets improved the strength which was due to micro inertia in the core design and also bending of interlayers was observed which led to an increase in energy absorption reducing the force oscillations [6]. Aluminum alloyed trapezoidal cores with fibre facesheets were investigated under different indenter geometries for their energy absorption, damage modes and it was reported that sharp tip indenters required high impact energy and damage modes were found to be matrix cracking with delamination of the facesheet [7]. Investigations on pyramidal truss structures by quasi-static indentation [8] and shaker mechanism showed that these structures are weak at their nodal points leading to disjoining of core and facesheet [9].

Apart from core design, failure mode maps prove to be very efficient in optimizing the sandwich parameters and improving their structural performance. Analytical models predicting failure modes such as face sheet failure, skin wrinkling, and core failure in relation to the applied force were developed and validated [10]. These models are useful in developing sandwich structures as per the requirements. Validation tests through finite element analysis on honeycomb sandwich panels showed that crack initiation was a direct result of localized deformation, and is independent of facesheet configuration. The effect of facesheet was found vital in contributing to failure maps [11]. Apart from experimental and numerical analysis, analytical models to map the failure modes were also developed to describe the response of bending deformation in plane strain conditions [12]. Shear failure arises in the core due to the result of bending moment in the core via the applied load and leads to core failure [13]. These shear failure modes can be controlled based on proper selection of facesheet material and orientation, as the inertia of the sandwich panel increases causing it to perform well under high loads [14,15]. A wide range of testing has been done on sandwich panels by varying the facesheet [12], the core materials [16] such as aluminum foam [17], the thickness of the core (thin walled structures) [18,19] and core designs such as circular composites [20], corrugated Y-frame cores [21], and bulk glass alloys [22]. Previously, work was done to report the role of various indenter geometries during indentation together with their energy absorption characteristics [17,23,24]. It was reported that sharp tip indenters require more energy to indent than standard hemispherical and a few other geometries. Therefore, it is evident that the indenter geometry has a great influence on the energy characteristics of the specimen.

Due to manufacturing limitations, there is a restriction in core design. Additive manufacturing (AM), is gaining popularity due to its compact manufacturing method of complex components and it is one of the most advanced manufacturing technologies. AM technology offers the ease of producing cost effective complex structures for various applications including tissue scaffolds for tissue engineering [25], bio printing of cell-hydrogels [26], lattice structures in orthopedic implants [27], heat sinks [28], and spacers in water treatment applications [29]. Three-dimensional (3D) printing may be one of the future manufacturing technologies that will serve the purpose of manufacturing complex parts for aerospace and defense. The applications of AM have been widely reported in aerospace and many industrial areas due to its advantages like complexity and variety free, no assembly requirement, little lead time, and less wastage of materials compared to the traditional machining process [30,31]. It was also reported that parts produced from AM will have long term sustainability in the aerospace industries [32]. 

Sandwich structures manufactured using AM technology proved to be lightweight, having high stiffness to weight ratio and shape recovery effect. Circular honeycomb core designs proved to have higher compression strength and the triangular cellular cores had a much faster recovery rate [33,34]. Investigation on out-of-plane compressive loading of 3D printed trapezoidal sandwich structures showed that the major failure mode was the buckling of vertical pillars [35]. Experimental investigation on compressive strengths of 3D printed sine wave and trapezoidal structures showed that trapezoidal structures had 12.12% higher compressive modulus than sine wave structures [36]. Sandwich structures printing using Inkjet printing technology led to performance evaluation of the ProJet^®^ multi material jetting 3D printer. Accuracies up to 13 µm were reported while printing [37]. Controlled printing for excellent strength-to-weight ratio was also developed and tested by printing hollow models [38]. 

Therefore, in this research work, inkjet printing technology using 3D systems’ ProJet^®^ MJP 5500X (3D systems, Rock Hill, CA, USA) was used to manufacture the sandwich panels. Two types of trapezoidal sandwich structures were printed; one with single material and uniform and the other with layering of two different materials. Later, the panels were tested for their indentation behavior under three different indenter geometries namely hemispherical, conical, and flat faced indenters. These indenters were selected to determine the failure mode of sandwich panels under various impacting objects apart from the standard hemispherical indenter. The effect of varying the facesheet material on the load bearing capacity of the structure, the energy absorption characteristics under each indenter, and the failure modes are here reported.

## 2. Three-Dimensional (3D) Printing of Core

### 2.1. Materials

The following work illustrates the lightweight structures developed for the aerospace industry. The trapezoidal sandwich structures were manufactured with the wide variety of materials offered from inkjet printing technology 3D Systems’ ProJet^®^ MJP 5500X. 3D Systems’ ProJet^®^ MJP 5500X has the capacity to mix materials and print them as composites. 3D Systems’ ProJet^®^ MJP 5500X materials include VisiJet^®^ CF-BK black rubber-like elastic material, and VisiJet^®^ CR-WT white rigid acrylonitrile butadiene styrene (ABS)-like material. One such combination was VisiJet^®^ RWT-FBK 400 slightly flexible material. Material properties of all the materials used are tabulated in Table 1.

Inkjet printing technology was used to manufacture the trapezoidal sandwich structure in the following combinations.

**Figure 1:**
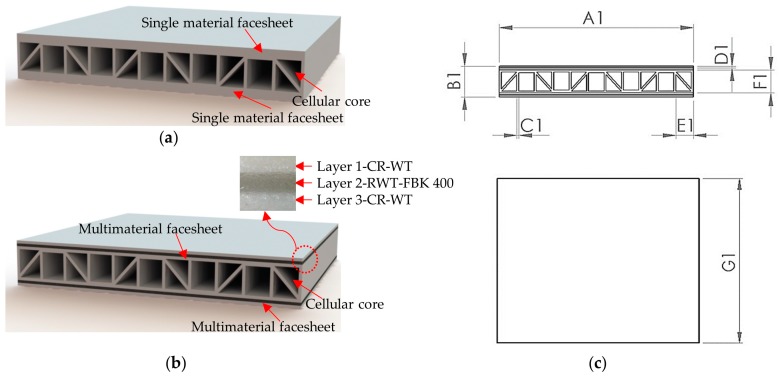
(**a**) Type A series specimen; (**b**) type B series specimen; and (**c**) sandwich panel nomenclature.

Type A: Both core and facesheet consist of VisiJet^®^ CR-WT white rigid ABS-like material as a single layer as shown in Figure 1a. Type B: Facesheet consists of three layers, Layer1-VisiJet^®^ CR-WT, layer 2-VisiJet^®^ RWT-FBK 400, and Layer 3-VisiJet^®^ CR-WT materials respectively as shown in Figure 1b.

Lightweight vertical pillared trapezoidal structures (vertical pillars were provided to enhance load bearing capacity during out-of-plane compression loading and 45 degree pillars were provided to support the vertical pillars to resist shear failure) with design specification as shown in Figure 1c with different material combinations were manufactured. The design specification of the structure designed is given in Table 2. The above trapezoidal geometry is widely used in aerospace and food packaging industries [39]. In this work, these structures were adopted to ensure their viability in the aerospace industries when manufactured using 3D printing technology. 

Specimen nomenclature is given in Table 3. For example, A-Hemi represents sandwich panel with VisiJet^®^ CR-WT white rigid ABS-like material and tested using a hemispherical indenter.

### 2.2. Processing of 3D Printed Sandwich Structure

**Figure 2:**
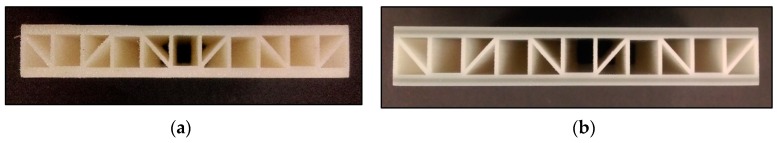
(**a**) 3D printed specimen with VisiJet^®^ CR-WT white rigid acrylonitrile butadiene styrene (ABS)-like material for core and facesheet; and (**b**) 3D printed specimen facesheet with three layers; Layer 1-VisiJet^®^ CR-WT, Layer 2-VisiJet^®^ Layer 2-RWT-FBK 400, and Layer 3-VisiJet^®^ CR-WT materials respectively.

The specimens used for quasi-static indentation were designed using computer-aided design (CAD) software—Solid Works 2015 (Dassault Systèmes, Vélizy-Villacoublay, Paris, France). The design was exported as Standard Tessellation Language (STL) file format and imported into the machine. Materials were assigned to various parts of the model created. Printing orientation was then assigned, as the printing direction plays a major role in determining the mechanical behavior and properties of the material. Upon completion of the printing, post processing was done on the 3D printed components to remove the wax support material. The wax was then removed in an oven by maintaining 65 °C for about 2–5 h. The specimens were then immersed in an oil bath to further remove the wax from the intricate structures. The samples were ready after final washing with water and drying. Figure 2 shows the final 3D printed specimens.

## 3. Test Procedures and Observations

### 3.1. Significance of the Test

Damage in the out-of-plane region due to concentrated force is a major concern in many light weight and laminated composite structures. During product development, the design and material selection of the composites and laminates are very useful. Quasi-static indentation testing is mainly carried out to study the force-displacement relationship of large mass impacts with a very small support region. An estimate of the energy required to impart damage to the structure is also calculated. The standard testing procedures include a rigidly backed condition in which the deflection effect of the specimen is restricted and an edge supported condition in which the specimen is placed on a plate having a circular opening and tested. In this case the specimen can deflect hence the deflection and stiffness effect can be considered.

### 3.2. American Society for Testing and Materials (ASTM) Standard

The test procedure as mentioned in ASTM D6264/D6264M-12 standard test method [40] for measuring the damage resistance of fiber-reinforced polymer matrix composite to a concentrated quasi-static indentation was used for analysis. The standard allows testing to be done under two different conditions. (1) Rigidly backed condition—suitable for specimens with rigid core and facesheet; and (2) edge supported condition—suitable for specimens with flexible or multi-material facesheet with the specimen deflection considered. In this work, the specimens were tested in an edge supported condition so that the effect of deflection of the specimen was considered and the indenter could penetrate until it indented the bottom of the specimens.

### 3.3. Experiment Equipment and Load

**Figure 3:**
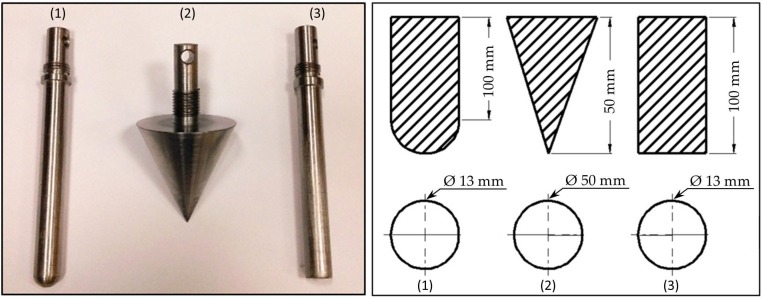
(**a**) Various indenters used for indentation experiment; and (**b**) geometrical dimension of indenters 1–Hemispherical indenter, 2–Conical indenter, 3–Flat faced indenter.

The tests were carried out in a universal testing machine SHIMADZU AG-X (Shimadzu Corporation, Kyoto, Japan) up to 10 kN. The fixture for testing, i.e., a plate made from aluminum with a thickness of 40 mm and a circular opening at the center with a diameter of 76.4 mm was used. A standard indenter with hemispherical tip and a diameter of 13.0 mm as per standards was used for the testing. Two other indenters were also used to study the effect of different indenter shapes during testing. Indenter shapes as shown in Figure 3 consist of a circular indenter with a hemispherical tip, a conical indenter with a sharp tip, and a circular indenter with a flat tip. During testing the indenter is aligned with the center of the specimen of offset at no more than 0.01 mm and then indented. Before starting the test, the indenter was made to slightly touch the top facesheet of the specimen and then a displacement of 1.25 mm/min was applied as per ASTM D6264/6264M-12. Quasi-static indentation being a displacement controlled phenomenon acts similarly like a low velocity impact. However, the end damage to the specimen can be studied only in impact testing. Force vs. displacement curves were obtained for all the tested specimens and then analyzed.

**Figure 4:**
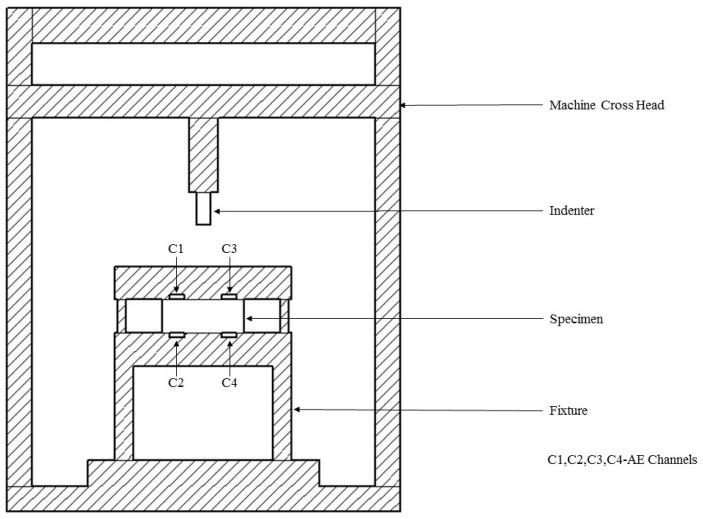
Machine setup-SHIMADZU AG-X up to 10 kN, along with acoustic emission sensors.

To measure the exact crack initiation and crack propagation in the specimen during brittle and quasi-brittle failure modes in indentation, a product of Vallen Systeme GmbH (Schaeftlarner Weg Icking, Germany), acoustic emission (AE) sensors an non-destructive testing (NDT) technique was incorporated. Vallen AMSY-6 data logger is equipped with eight AE channels and 10 parametric channels. However, only four AE channels were utilized during this test. Four AE sensors (100–450 KHz, resonance at 150 kHz) were mounted at the top and bottom of the fixture as shown in Figure 4 and the peak amplitude vs. time was obtained. 

Sandwich panels are prone to different damage modes based on the geometry of the impact body. Thus, studying the effect of quasi-static indentation under an indenter with hemispherical indenter is not enough. Hence indenters with three different geometries were used in the study and the effect of indenter geometry studied. 

### 3.4. Experimental Plan

**Figure 5:**
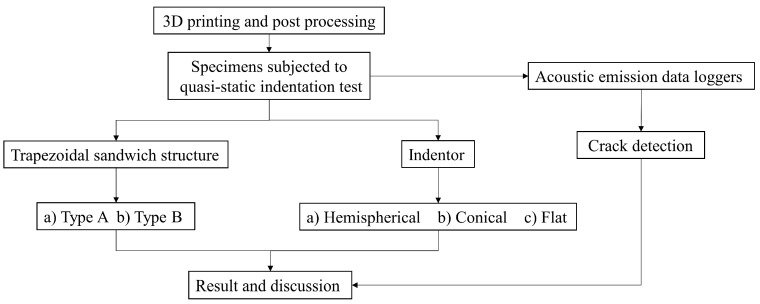
Experimental plan.

The experimental plan is shown in Figure 5. As discussed specimens were 3D printed with two different combinations of materials as per type A and type B in Section 2.1. The specimens were then subjected to quasi-static indentation testing along with AE data loggers for capturing invisible and minor cracks inside the specimen. In each case, five specimens were tested and their average result is tabulated in Section 4. Finally, results are discussed based on the effect of 3D printed materials, the effect of indenter geometry and crack initiation, as well as crack propagation timing using AE data logger readings.

## 4. Result and Discussion

A quasi-static indentation force was applied on the specimen using displacement controlled phenomenon and data recording was done at a sampling rate of 5 data per second. The indenter was made to fully penetrate inside the test specimens and then the damage was observed on both the top and bottom side. All the compliance changes and damage were noted during testing. AE sensing was also used to monitor minor cracks in the specimen. The force vs. displacement, force vs. peak amplitude and peak amplitude vs. time graphs during testing were represented as follows

### 4.1. Force vs. Displacement, Force vs. Time and Peak Amplitude Pattern under Hemispherical Indenter

**Figure 6:**
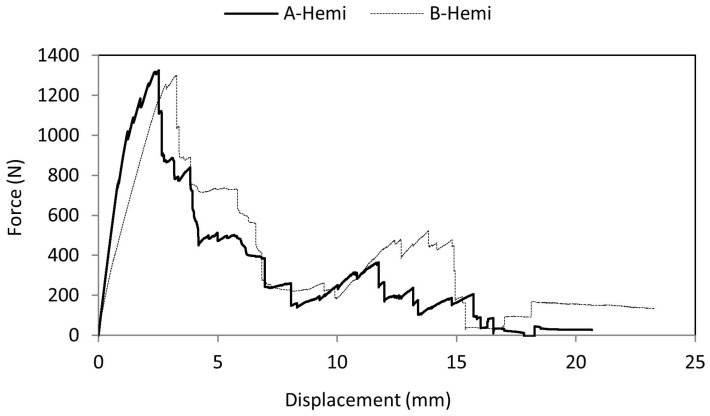
Comparison of the force-displacement curve of A-Hemi and B-Hemi specimens.

Figure 6 shows the average force-displacement curve for A-Hemi and B-Hemi series of the specimen. It was found that there was a rise in force values up to 1.36 kN until an indenter displacement of 2.62 mm after which the crack was initiated in the top facesheet and failure occurred. The type of failure mode observed was purely a brittle failure. This brittle failure of the top facesheet was due to the rigid material nature. Due to the core design (due to the trapezoidal angular wall and vertical pillar resistance to indenter penetration), there was an intermediate rise and drop of force values as the indenter progressed further. At a displacement of 12.5–13 mm there was again a rise in force values. This was the effect of bottom facesheet indentation after which the specimen failed completely by a sudden crack with brittle failure and the force values dropped to zero.

B-Hemi series specimens failed only after a displacement of 3.37 mm while the A-Hemi series specimen failed at 2.39 mm. The elastic nature of the material caused the specimen to deflect in the axial loading direction which indirectly led the indenter to penetrate more into the specimen. During indentation, top facesheet failure was observed as quasi-brittle. This failure nature was due to the facesheet material configuration, as top and bottom ply were made of rigid ABS-like material and the mid ply was made of rubber-like elastic material.

**Figure 7:**
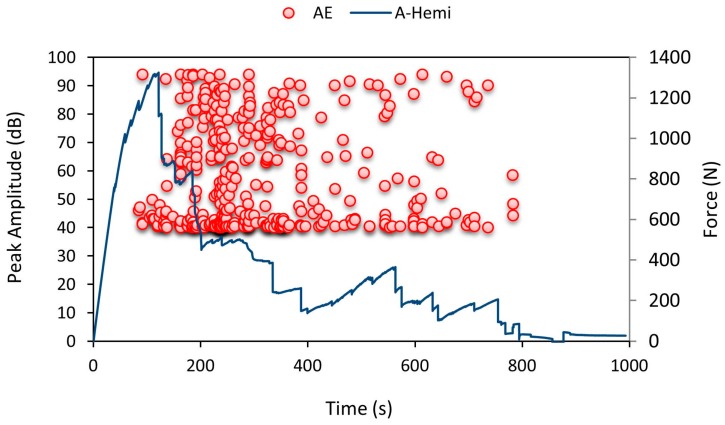
Average force-time curve for A-Hemi series with recorded acoustic emission (AE) pattern.

AE sensors were mounted to capture minor brittle crack timing throughout indentation, from Figure 7 it is noted that the number of AE hits was higher over 100–120 s which clearly shows that the specimen experienced a higher number of AE hits (minor internal crack recorded by AE data loggers) and the top facesheet failed. It is also evident that until 120 s the amplitude was very low and during failure at 120 s the amplitude values rose higher in all the four channels which denoted brittle failure in the specimen with a loud noise.

**Figure 8:**
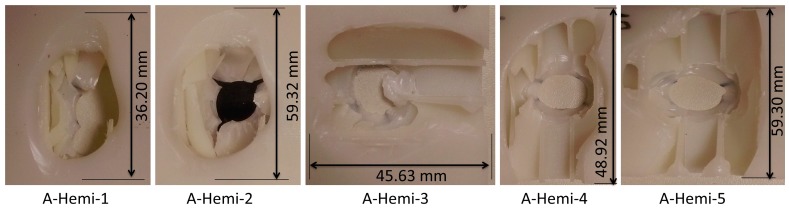
Observed damage modes of out-of-plane loading of A-Hemi series specimens.

Five specimens were tested in each case and their damage geometry on the bottom facesheet is shown in Figure 8. The damage shapes were mostly found to be circular and rectangular. The area of damage made by the indenter was measured using ImageJ software (open source Java image processing program) [41,42]. These damage areas were then used to compute the percentage of damage occurring in the specimen. The hemispherical indenter made 33.4% damage to the specimen. On the top facesheet, the damage shape was found to be only circular and its magnitude was directly related to the diameter of the indenter.

**Figure 9:**
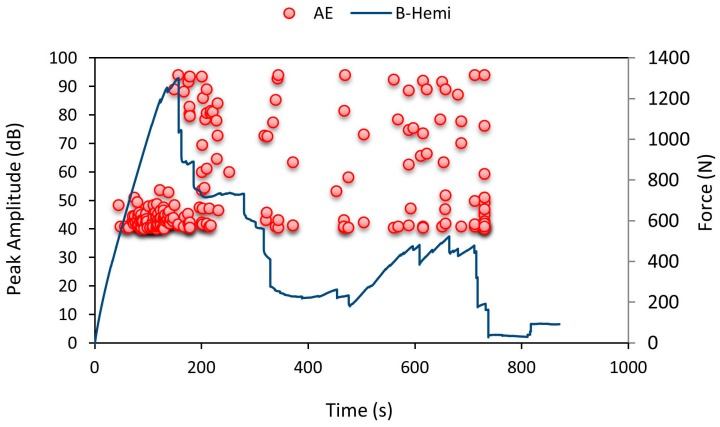
Average force-time curve for B-Hemi series with recorded AE pattern.

From Figure 9, from 50 s to 180 s, crack initiation occurred with noticeable plastic deformation and delamination in the specimen and it can be concluded that the crack started to propagate around 180 s and a drop in force values was observed with a rise in amplitude due to the cracking noise. During bottom facesheet indentation, the force values seemed to rise higher due to the specimen deflection and then failed. The elastic ply in between the two-rigid ply in the facesheet was the reason for specimen deflection without failure during indentation. Even before failure, minor brittle cracks in the top and bottom rigid ply were recorded as AE hits in the data loggers. When the deflection exceeded the elastic limit, the specimen failed with a brittle fracture. Figure 9 clearly shows the bottom facesheet failure at 720 s during which the specimen experienced a higher number of AE hits with a high peak amplitude denoting failure.

**Figure 10:**
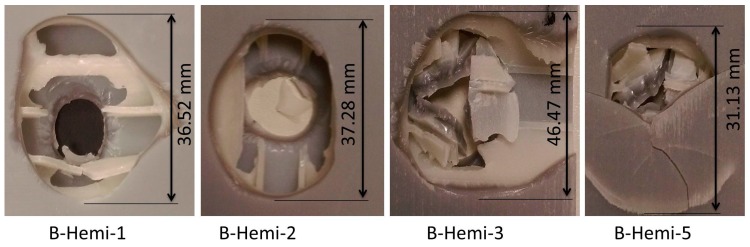
Observed damage modes of out-of-plane loading of B-Hemi series specimens.

The damage shapes induced by the hemispherical indenter on B-Hemi series specimens are shown in Figure 10. All the shapes were found to be circular on both the bottom and top facesheets. The damage made was 30.04% and was lower compared to the A-Hemi series specimens. Due to the brittle nature of the material used, A-Hemi series specimens had larger cracks in them leading to higher damage percentage. While B-Hemi series specimens with a RWT-FBK 400 layer absorbed the energy leading to less crack and lower damage percentage. 

### 4.2. Force vs. Displacement, Force vs. Time and Peak Amplitude Pattern under Conical Indenter

**Figure 11:**
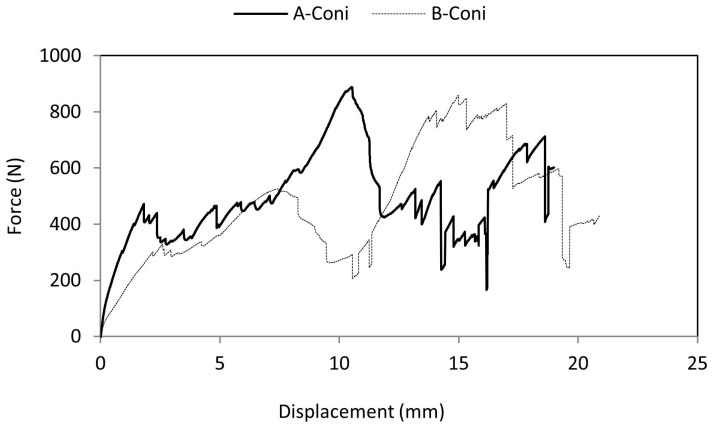
Comparison of force-displacement curve of A-Coni and B-Coni specimens.

Figure 11 represents the force-displacement curve when indented using a conical indenter. Behavior was entirely different from the previous case. Due to the sharp tip of the indenter, brittle failure was observed in the top facesheet and failure occurred without rise in force values. When the indenter progressed further, due to the surface contact between the indenter walls and the specimen, there was a continuous rise in force values and a drop in force values due to the brittle core cracking. A drop in force values was recorded at 12.03 mm indenter displacement for A-Coni series and 12.84 mm for B-Coni series specimens. Both the A-Coni and B-Coni series could withstand an average of 920 N and 766 N during failure irrespective of the material used for the facesheet. The load bearing capacity was significantly influenced by the indenter geometry.

**Figure 12:**
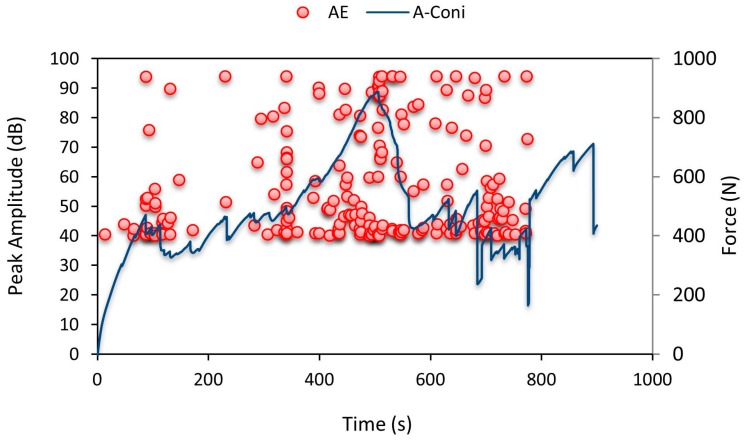
Average force-time curve for A-Coni series with recorded AE pattern.

Figure 12 shows the AE pattern recorded. Until 90 s there were minor cracks initiated in the specimen and after that, top facesheet failure occurred at around 90 s. Until 90 s the amplitude values recorded were only 40 dB noise level. After which they were raised to 95 dB denoting cracking in the top facesheet. From 300 s to 660 s there was a rise and fall of force values along with high amplitude data points recorded by AE data loggers denoting crack propagation throughout the specimen. This pattern continued until specimen failure.

**Figure 13:**
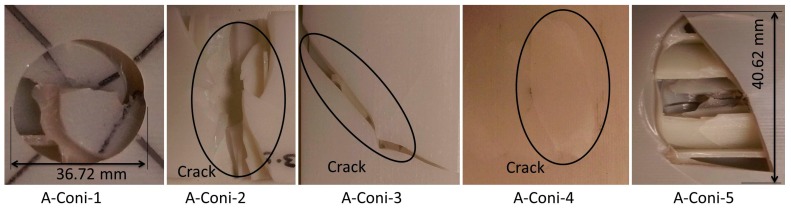
Observed damage modes of out-of-plane loading of A-Coni series specimens.

The damage geometries of the A-Coni series specimens under a sharp conical indenter as shown in Figure 13 were found to be circular, rectangular, and triangular. Specimens even failed leaving over few cracks in the bottom facesheet with force values reducing to zero. The damage due to the conical indenter was 48.62%. This is higher compared to the A-Hemi series specimens and is purely because of the indenter geometry. In some cases, the specimens failed even before the indenter fully penetrated through the bottom side of the facesheet, while hemispherical indenters fully penetrated inside. 

**Figure 14:**
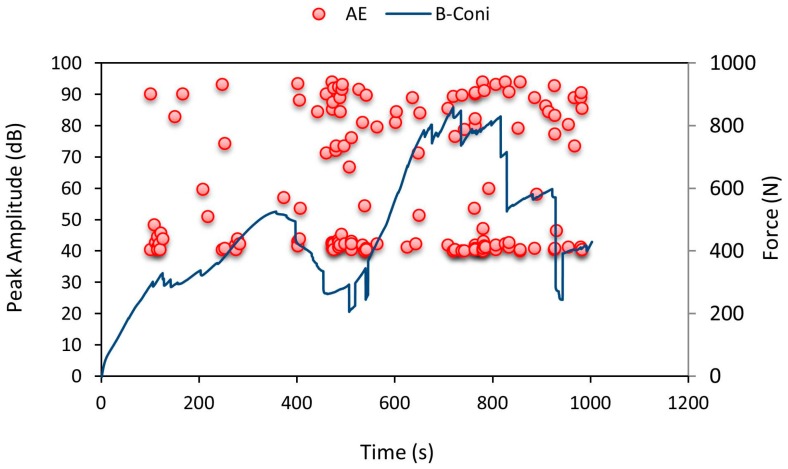
Average force-time curve for B-Coni series with recorded AE pattern.

AE pattern for B-Coni series of the specimen is shown in Figure 14. From the amplitude values obtained using an AE data logger, it can be observed that the values of noise amplitude were very high around 95 dB from the beginning of the experiment, which represents cracking in the specimen and a cluster of data was recorded over 400–450 s representing brittle crack in the specimen with a drop in force values. The force values seemed to increase continuously throughout the experiment until the specimen cracked into two halves.

**Figure 15:**
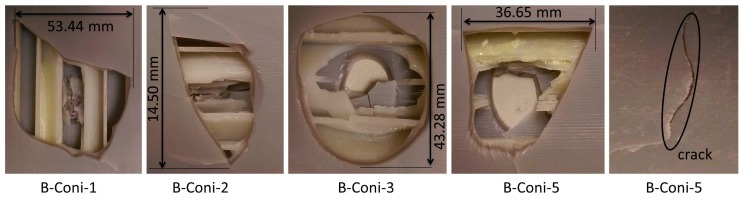
Observed damage modes of out-of-plane loading of B-Coni series specimens.

The damage shapes of B-Coni series of specimens as shown in Figure 15 were found to be square, circular, and triangular, with few cracks on the bottom facesheet. Damage of 36.50% was noticed for the conical indenter in the B-Coni series and this was due to the sharp tip of the indenter that pierced through the bottom facesheet. Due to the elastic nature of material used in the B-Coni series, more energy was absorbed making the indenter penetrate further down to impart damage to the specimen which led to a higher damage percentage compared to the A-Coni series specimens.

### 4.3. Force vs. Displacement, Force vs. Time and Peak Amplitude Pattern under Flat Indenter

**Figure 16:**
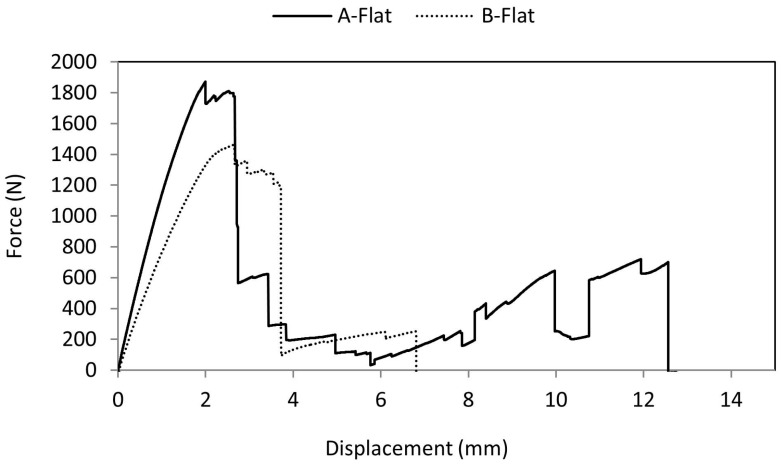
Comparison of force-displacement curve of A-Flat and B-Flat specimens.

When a flat indenter was used for indentation, the force-displacement curves as shown in Figure 16 were found to be different from the other two cases. The rise in force values was seen until 130 s and due to further loading, the force values dropped suddenly and again started to rise until the bottom facesheet failed. The failure was purely due to brittle fracture as the specimens immediately cracked without plastic deformation.

**Figure 17:**
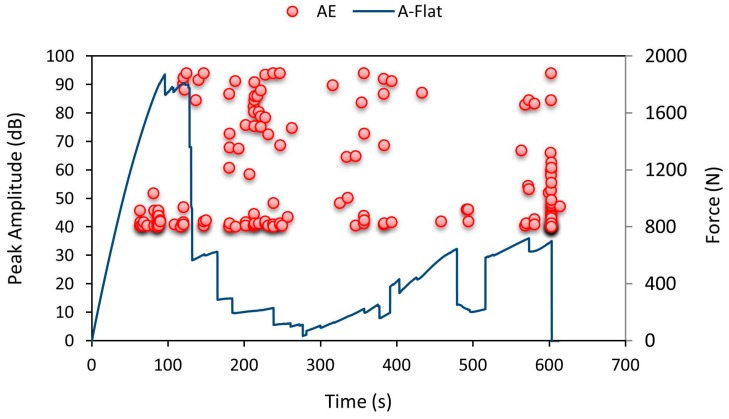
Average force-time curve for A-Flat series with recorded AE pattern.

As the indenter started to penetrate the specimen, at 90 s low amplitude values were recorded showing minor crack initiation in the top facesheet. Figure 17 shows that the number of AE hits was higher at 120–230 s during which there was a crack initiation in the specimen and intermediate rise and fall in force values were noted as an effect of core design; the specimen failed around 570–600 s. From the amplitude values recorded using AE data loggers, there was rise in amplitude over 90–100 s and at 570 s respectively. These amplitude patterns gave us the information to confirm the brittle crack initiation and specimen failure timings accurately.

**Figure 18:**
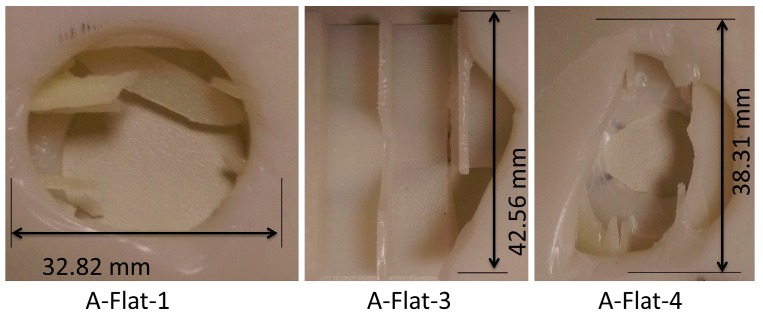
Observed damage modes of out-of-plane loading of A-Flat series specimens.

Damage geometry in A-Flat series of specimens as shown in Figure 18 was found to be rectangular and circular. Few specimens even cracked into two halves. A damage of 40.94% was made by the flat indenter. The damage modes during failure were found to be brittle cracking of the core.

**Figure 19:**
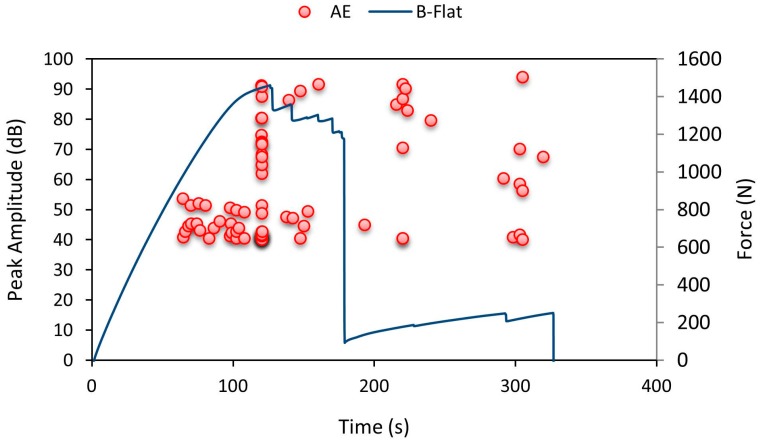
Average force-time curve for B-Flat series with recorded AE pattern.

B-Flat specimens were found to fail with a drop in force values as shown in Figure 19. The specimens mostly failed before the indentation of the bottom facesheet. Due to the flat indenter, the specimens buckled and due to further displacement of the indenter, the specimens were found to fail with a brittle fracture. As shown in Figure 19, low amplitude AE data values were recorded from 70 to 120 s specifying minor crack in the specimen. A rise in the number of AE hits was observed at 80 s. The amplitude pattern shows that there was a rise in amplitude value around 120 s and this pattern continued until the specimen failed.

**Figure 20:**
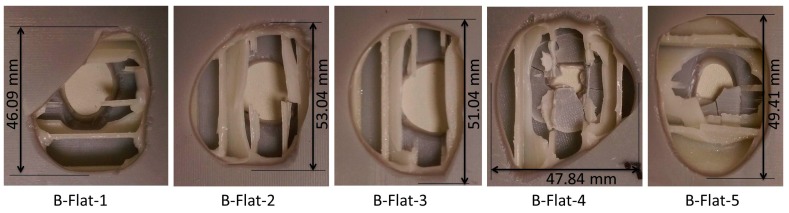
Observed damage modes of out-of-plane loading of B-Flat series specimens.

Damage geometry on the bottom facesheet of B-Flat series of specimens as shown in Figure 20 was only of the circular type. Even the top facesheet incurred only circular damage. Damage of 36.16% was observed. The flat indenter completely penetrated through the bottom facesheet leaving the damage shape to be circular, the same as that of its contact face.

### 4.4. Energy Absorption Characteristics

The total energy absorbed by the specimen during indentation is dependent on the indenter geometry and type of material used for the specimen. The energy absorbed by the specimen is calculated from the area under the force-displacement curve as specified in ASTM D6264/6264M-12. Area under the curve is calculated by using

$$E_{\max}=E\left(\delta_{\max}\right)=\int_{\delta_{0}}^{\delta_{\max}}F\left(\delta \right)d\delta$$

*E*_max_ represents the energy required for the indenter to reach its maximum displacement. This energy also represents the energy required to impart failure in the specimen. The failure mentioned here denotes that the specimen’s bottom facesheet is indented, the specimen has cracked into halves or the force value has reached zero.

From the energy calculation, it can be concluded from Table 4 that the energy required to reach maximum displacement is 9.40 J with a conical indenter for A-Coni series specimens and about 9.66 J for B-Coni series specimens. Indentation energy required for a flat indenter to indent was about 6.96 J for A-Flat series and 8.95 J for B-Flat series. Therefore, the hemispherical indenter required the lowest energy to make an indentation which was about 6.87 J and 8.82 J for A-Hemi and B-Hemi series specimens respectively. This may be because when the conical indenter indented downwards, there was reduced resistance to indent the top facesheet as the indenter has a very sharp tip. As it progressed further the contact between the indenter and the specimen increased due to the conical geometry. The contact between the indenter and specimen is the main reason for the higher energy required by the indenter to displace into the specimen. It is also true in the case of a flat and hemispherical indenter, the energy required is higher for the flat indenter as there is a huge contact area between the indenter and the facesheet. In the hemispherical indenter, the contact area is only a single point due to the curvature of the indenter tip. In short, the energy required for displacement is lower. Therefore, indenter geometry and contact area have a major influence on the energy required for maximum displacement. When the contact area increases, the energy required for maximum displacement increases.

The reasons for the high standard deviation in the displacement values in Table 5 with the conical indenter are as follows: Indenter displacements in the above mentioned three possible modes of failure are entirely different, it is evident that in (1) the indenter penetrated through the bottom facesheet, therefore the displacement of the indenter at failure is equal to or greater than the thickness of the specimen (displacement ≥16 mm); in (2) the indenter did not penetrate the bottom facesheet (position of indenter is inside the core), but the specimens failed (cracked into two halves) due to crack propagation with force values reaching zero, therefore the displacement of the indenter at failure is less than the specimen thickness (3 mm < displacement < 16 mm); and in (3) the force values reached zero after the indenter penetrated the top facesheet forcing the specimen to crack, therefore the displacement of the indenter at failure is equal to or less than the top facesheet thickness (displacement ≤3 mm). 

## 5. Conclusions 

The indentation behavior of a 3D printed vertical pillared trapezoidal sandwich structure was studied by quasi-static indentation testing under edge supported conditions. The indentation effect by varying the facesheet material was also discussed. Special emphasis was given to the damage behavior by varying the indenter geometry. Some of the major findings include:

(1) Indenter geometry (geometry of impacting object) has a major influence in determining the load bearing capacity of the sandwich structures. Type A and type B series specimens could withstand loads up to 2.0 kN and 1.54 kN under a flat faced indenter. The specimens are prone to damage easily under sharp tip indenters with a low load bearing capacity of 0.92 kN and 0.76 kN for Type A and type B series specimens. These findings also agree with the findings of other researchers.
(2) 3D printed trapezoidal sandwich structures of type A series can withstand high loads but fail immediately with brittle behavior with lower indenter displacements. This failure mode was because of the brittle material used for 3D printing. In addition, the type B series specimens could only withstand low loads but failed only after higher indenter displacement. Indenter geometries and facesheet material variation were found to be the factors most influencing the load bearing capacity and energy absorption characteristics of the specimen.
(3) High sensitive AE patterns as a cluster of low amplitude noise levels indicate invisible crack initiation in the specimens and high amplitude noise levels obtained indicate crack propagation in the specimen. Therefore, this type of high sensitive crack detection technique is useful in detecting even minor and internal failures in aerospace applications reducing the high risk of complete part failure during operation.

## Figures and Tables

**Table 1:** Properties of materials used for manufacturing sandwich panels. ABS: acrylonitrile butadiene styrene.

Material/Property	VisiJet^®^ CR-WT White Rigid ABS-Like Material	VisiJet^®^ CF-BK Black Rubber-like Elastic Material	VisiJet^®^ RWT-FBK 400 Slightly Flexible Material
Density (g/cc)	1.04	1.04	1.04
Tensile Strength (MPa)	56	2.2	10
Elongation at Break (%)	8.1	290	25
Flexural Strength (MPa)	74	0.5	7.3

**Table 2:** Design specification of sandwich panel.

Nomenclature	A1	B1	C1	D1	E1	F1	G1
Vertical pillared trapezoidal structures (All dimensions are in mm)	100	16	1	1	10	12	100

**Table 3:** Specimen nomenclature.

Nomenclature	Definition
Type A	Specimen facesheet with VisiJet^®^ CR-WT white rigid ABS-like material
Type B	Specimen facesheet with three layers Layer 1-VisiJet^®^ CR-WT, Layer 2-VisiJet^®^ Layer 2-RWT-FBK 400 and Layer 3-VisiJet^®^ CR-WT materials respectively
Hemi, Coni, Flat	Testing was done using hemispherical, conical, and flat indenters respectively

**Table 4:** Maximum energy required by the indenter for displacement.

Specimen	*E*_max_ (J)
A-Hemi	6.87
A-Coni	9.40
A-Flat	6.96
B-Hemi	8.82
B-Coni	9.66
B-Flat	8.95

**Table 5:** Experimental observation of sandwich panels.

Specimen	Max Force that the Specimen Can Withstand during Crack Initiation	Max Displacement During Drop in Force	Damage Area on Top Facesheet	Damage Area on Bottom Facesheet	Percentage of Damage Occurred in Exposed Area (Bottom Side)
	(kN)	(mm)	mm^2^	mm^2^	(%)
A-Hemi Average ± SD	1.36 ± 0.07	2.62 ± 0.40	133.60	1531.00	33.40
A-Coni Average ± SD	0.92 ± 0.21	12.03 ± 2.02 *	168.28	396.12	48.62
A-Flat Average ± SD	2.0 ± 0.29	2.34 ± 0.31	132.02	963.32	40.94
B-Hemi Average ± SD	1.31 ± 0.16	3.37 ± 0.38	133.41	1379.20	30.04
B-Coni Average ± SD	0.76 ± 0.29	12.84 ± 5.15 *	163.43	757.80	36.50
B-Flat Average ± SD	1.54 ± 0.13	2.25 ± 0.50	133.30	1659.16	36.16

* Specimens under conical indenter failed in three possible modes (1) Bottom facesheet failure after indenter penetration; (2) Specimen cracked into two halves during indenter penetration with force values reducing to zero; and (3) Force values reached zero during indenter penetration on top facesheet (specimens did not crack and indenter did not penetrate through bottom facesheet).
